# Quantitative Assessment of Full Size Microplastics in Bottled and Tap Water Samples in Hong Kong

**DOI:** 10.3390/ijerph192013432

**Published:** 2022-10-18

**Authors:** Yuet-Tung Tse, Sidney Man-Ngai Chan, Eric Tung-Po Sze

**Affiliations:** School of Science and Technology, Hong Kong Metropolitan University, Hong Kong Special Administrative Region, Hong Kong, China

**Keywords:** microplastics, bottled water, tap water, flow cytometry, Nile red fluorescence staining, visual identification, microplastics size distribution

## Abstract

Human exposure to microplastics (MPs) through drinking water has drawn serious concern recently because of the potential adverse health effects. Although there are reports on the occurrence of MPs in bottled water, little is known about the abundance of a whole spectrum of MPs with sizes ranging from 1 µm to 5 mm due to the restrictions of conventional MPs detection methods. Some studies using micro-Raman spectroscopy can achieve MPs with a size of <10 µm, however, quantitation of all MPs was extremely time consuming and only a small portion (<10%) of MPs would be analyzed. The present study quantified MPs from nine brands of bottled water using fluorescence microscopy and flow cytometry for MPs with a size of ≥50 µm and a size of <50 µm, respectively. The average abundance of MPs with a size of ≥50 µm in bottled water samples was found ranging from 8–50 particles L^−1^, while MPs with a size of <50 µm were found to be 1570–17,817 particles L^−1^, where the MPs abundance from mineral water samples were significantly more than distilled and spring water samples. The modal size and shape of MPs were found at 1 µm and fragments, respectively. Besides, three tap water samples obtained locally were analyzed and compared with the bottled water samples, where less MPs were found in tap water samples. In addition, contamination of MPs from bottle and cap and interference by addition of mineral salts were studied, where no significant difference from all these processes to the control sample was found, suggesting the major contamination of MPs was from other manufacturing processes. Estimated daily intake (EDI) of MPs increased substantially when data of small MPs are included, suggesting that previously reports on exposure of MPs from drinking water might be underestimated, as only large MPs were considered.

## 1. Introduction

The issue of microplastics (MPs) pollution has received increasing attention from the scientific community, society, and the public. Due to the continued mass production of plastic products and mismanagement of plastic wastes, microplastics [1,2], which are defined as plastic particles with sizes ranging from 1 µm to 5 mm, are ubiquitous not only in different environmental compartments, such as marine water [3], sediment [4], and biota [5], but also in human foods, beverages and drinking water [6,7]. Although the toxicity of MPs to humans remains unclear, a growing body of evidence suggests that MPs can cause physical obstruction in the gastrointestinal system and hence accumulate in the human body [8], while MPs with a smaller size might exert additional toxic effects. For example, an in vitro study from Hwang et al. [9] reported that polypropylene with a diameter of less than 20 µm would affect the production of the cytokine IL-6 and cause early stage inflammation. MPs with a size of less than 5 µm could enter into bloodstream and induce hemolysis of red blood cells, whereas MPs with a larger diameter (>10 µm) had no impact. Goodman et al. [10] demonstrated that MPs at 1 µm size would decrease metabolic activity of human lung cells up to 50% and reduce proliferative ability. The results suggested that MPs not only have the potential to cause physical effects in humans, but also induce cellular and metabolic toxicity when the size of MPs is decreased.

Due to being cheap in price and unstable water supplies, bottled water has become an important source of drinking water worldwide. For example, as of 2022, the revenue of bottled water in China is expected to reach USD 68.82 billion, with an annual growth rate of 6.56%, and the annual average volume of bottled water consumed per person would reach 76.6 L, demonstrating that there is a significant demand for bottled water [11]. Several studies have reported MPs contamination in bottled water. Zhou et al. [12] analyzed 23 brands of bottled water from the market of China, where they found an average of 16 particles per L^−1^ of MPs with a size larger than 25 μm. Another study detected an average concentration of 325 and 10.4 particles per L^−1^ for MPs with a size of 6.5–100 μm and ≥100 μm, respectively, in bottled water from nine countries [13]. However, most of the available studies on MPs occurrence in bottled water mainly focused on MPs larger than 5 µm, with only a few studies that reported MPs contamination levels with sizes down to 1 µm [14,15].

Optical microscopy is commonly used to assess MPs in water samples based on morphological characteristics, such as particle shape and color. The identities of the discovered particles can then be confirmed using spectroscopic techniques, such as Fourier Transform Infrared (FTIR). However, this method can only be used to characterize MPs greater than 100 µm [16]. Advanced instrumentation, such as micro-FTIR, can extend the analytical size range down to 10 µm, albeit at the cost of much longer measurement time [17]. As a result, it was a bit challenging for researchers to detect and quantify small MPs in water samples in the past. Until recently, we have developed an analytical method to pre-concentrate MPs in environmental water samples by vacuum filtration, to tag MPs with fluorescent staining, and to quantify small MPs sized from 1–50 µm using flow cytometry [18], which could be used in determining the complete spectrum of MPs from 1 µm–5 mm in conjunction with the optical microscopy technique.

In this study, a quantitative assessment was conducted to determine the full-size MPs contamination in nine different brands of bottled water obtained from the local market, including distilled water, natural spring water and mineral water, as well as three tap water samples, obtained locally. Large MPs (≥50 µm) were determined manually using fluorescence staining and microscopy, while fluorescence staining and flow cytometry were applied for small MPs (1–50 µm) quantification. The relationships between MPs abundance, size distribution and water types were analyzed. In addition, the contamination levels of MPs in bottled and tap water samples obtained in this study were further used for assessing human exposure to MPs. To our best knowledge, this is the first time full scale MPs from 1 µm to 5 mm in bottled and tap waters have been determined. These findings will contribute to a comprehensive profile of MPs abundance and size distribution in bottled and tap waters, and also the estimated daily intake of MPs through bottled and tap waters.

## 2. Materials and Methods

### 2.1. Materials and Sample Collection

Nine different brands of bottled water from different origins were purchased from the local market, which was composed of three distilled waters, three natural spring waters and three mineral waters. Table 1 summarized the characteristics of all samples, including brand, origin, water types, volume, price, and packing materials. Three tap water samples were collected from three different locations in Hong Kong (Wong Tai Sin (A), Ho Man Tin (B) and Tseung Kwun O (C)) with laboratory glass bottles and glass stoppers. Samples were stored at room temperature and analyzed within one week.

Except the polyethylene terephthalate (PET) powder (fragment type, with average size at 4.9 µm) purchased from Special Plastic Lang Chemical (Dongguan, China), all chemicals used were analytical reagent (AR) grade. Tween 20 was purchased from Santa Cruz Biotechnology, Inc. (Dalla, TX, USA). Ethanol (95%) was purchased from Honeywell Research Chemicals (Charlotte, NC, USA). Nile red (NR) was purchased from Signa-Aldrich Corp. (St. Louis, MI, USA). Ultrapure water was produced by a Milli-Q water system (Merck Millipore, Hong Kong, China). An NR stock solution (1 mg mL^−1^) was prepared by dissolving NR in 95% ethanol. The solution was stored at 4 °C in the dark until use.

### 2.2. Quality Control and Contamination Prevention

To avoid background contamination throughout the experiment, all glassware was washed and rinsed at least three times with ultrapure water and baked in a muffle furnace at 500 °C for 5 h to remove all residual organics and particles. All glassware was covered with aluminum foil before use to prevent the airborne particle contamination. NR stock solution and 5% Tween 20 (*w*/*v*) stock solution were filtered through 0.45 µm mixed cellulose esters (MCE) filters (Toyo Roshi Kaisha, Ltd., Tokyo, Japan) before use. Unless specified, samples were determined in triplicate and all the samples were handled inside a ductless fume hood (ESCO, Singapore) with laminar airflow.

### 2.3. Pre-Concentration of MPs and Fluorescence Staining

Pre-concentration of MPs and fluorescence staining of bottled and tap water samples was conducted with reference to the method specified in our previous study [18], except without digestion of organic carbon. Five liters of each sample was filtered through a stainless-steel sieve with a 50 µm mesh size, where particles with a size of ≥50 µm retained on the sieve were rinsed off with ultrapure water and collected in a beaker. NR stock solution was added to the rinsate to achieve an NR concentration of approximately 10 µg mL^−1^ for staining and the mixture was incubated for 10 min at ambient temperature. The mixture after staining was then filtered through a 0.45 µm MCE membrane filter with suction (vacuum pressure at 15 In. Hg), and the membrane filter was dried at 60 °C in an oven for 2 h prior to microscopic identification.

The filtrate after passing through the stainless-steel sieve with 50 µm mesh size was filtered under vacuum through another 0.45 µm MCE membrane filter. Particles with a size of <50 µm in the resulting MCE membrane filter were resuspended in a solution of Tween 20 (0.1% (*w*/*v*)) with mechanical action using a pair of metal tweezers. One milliliter of the solution was aliquoted into a 1.5 mL micro-tube (Eppendorf, Germany). Ten microliters of NR stock solution (1 mg mL^–^^1^) was added to the aliquot and incubated at room temperature for 10 min prior to the quantification.

### 2.4. Sample Quantification

Filters containing stained MPs with a size of ≥50 µm were identified using a stereomicroscope (Meiji Techno Co., Ltd., Saitama, Japan) equipped with a 450 nm blue LED light source (Spectroline OFK 8000A, Spectro-UV, Farmindale, New York, NY, USA) and an orange emission filter (Spectroline OFK 8000A, Spectro-UV, Farmindale, New York, NY, USA) at magnification of up to 45×. MPs were defined as those that produced red, yellow, or orange fluorescence light, with their shapes (e.g., fragment and fiber) recorded.

On the other hand, solutions containing stained MPs with a size of <50 µm were analyzed by flow cytometry (Bio-Rad ZE5 Cell Analyzer). A target laser at 488/10 nm and a filter of 583/30 nm (orange) were chosen for excitation and emission, respectively. The trigger was set to 0.01% forward scatter, and the photomultiplier tubes (PMT) voltages were set at 140 for forward scatter (FSC), 280 for side scatter (SSC) and 425 for 583/30 nm. The samples were analyzed at a flow rate of 0.4 µL per second and a total volume of 100 µL was analyzed for each sample. After each run, the sample line and probe were flushed by ultrapure water to minimize cross contamination. To distinguish between stained MPs and background signals with NR precipitates, a reagent blank composed of Tween 20 at 0.1% (*w*/*v*) in ultrapure water stained with NR was used to determine the background for correction. A set of polystyrene calibration beads (1, 4, 6, 10, 15 and 38 µm) were analyzed to calibrate the size of MPs identified.

To assess the efficiency in quantifying stained MPs in the presence of drinking water matrix by flow cytometry, a recovery study was conducted by spiking solution containing PET powder to the specimen before NR staining and flow cytometry determination. A spiking solution was prepared by adding approximately 0.65 g of PET powder in 15 mL of ultrapure water with 0.1% (*w*/*v*) Tween 20 to achieve a concentration of 42,900 ± 14,000 particle mL^–1^ (quantified by flow cytometry with NR staining, *n* = 5). Ten liters of distilled water (Sample #3) was filtered through a stainless-steel sieve with a 50 µm mesh size prior to a 0.45 µm MCE membrane filter. Particles with a size of <50 µm retained on the MCE membrane filter were resuspended in 20 mL of ultrapure water with 0.1% (*w*/*v*) Tween 20 to serve as the drinking water matrix. The drinking water matrix was aliquoted into 1 mL specimens, where the specimens were spiked with an equal volume of either ultrapure water (i.e., sample blank) or the spiking solution (i.e., sample spike). Both sample blank (*n* = 3) and sample spike (*n* = 3) then underwent NR staining, followed by flow cytometry quantification. Recovery was calculated by dividing the difference in the average amount of MPs found between the sample blank and sample spike with the average amount of PET spiked to the sample spike:

(1) $$\mathrm{Recovery}\;(\%)=\frac{\bar{MPs\;in\;sample\;spike}-\bar{MPs\;in\;sample\;blank}}{\bar{amount\;of\;PET\;spiked}}\times 100\%$$

### 2.5. Assessment of Potential MPs Contamination and Interference

To assess the effect of MPs contamination through plastic bottle filling and storage processes, empty single-use PET bottles (capacity one liter) (Yuancheng Plastic Products Co., Ltd., Zhengzhou, China) were pre-washed at least three times with ultrapure water, filled with ultrapure water to the point of overflowing, and sealed with a polyethylene tethered cap to mimic the bottle filling. Five of the filled bottles (with a total volume of five liters) were re-opened immediately after being capped and pooled as a sample to assess any contamination of MPs from the filling process. To assess any increase in MPs during storage, a set of five single-use PET bottles with tethered caps that pre-filled with ultrapure water were stored at ambient temperature for one month and pooled as a sample to assess any contamination of MPs during storage in the bottle. All the above-mentioned samples after being pooled were subjected to the sieving, pre-concentration, fluorescence staining and sample quantification, and the experiments were repeated in triplicate to determine the repeatability.

### 2.6. Assessment of Potential Interence by Mineral Salts Addition

To determine any interference to flow cytometry signal induced by the addition of salts in mineral water, bicarbonate (HCO_3_^−^), calcium (Ca^2+^) and sodium (Na^+^) salts at 3 different concentrations (380, 240 and 50 mg L^−1^) were dissolved individually into 10 mL ultrapure water with 0.1% Tween 20, and the resulting salt solutions were stained with NR to a final concentration of 10 µg mL^−1^, prior to the analysis of flow cytometry. The salt concentrations selected were based on information from the mineral water products’ ingredient labels.

### 2.7. Data and Statistical Analysis

Statistical analysis of the collected data in this study was performed by using GraphPad Prism 8.3.0. The mean and standard deviation of the triplicates from each experiment were calculated. To determine any significant difference between MPs concentration and bottled water types, one-way AVOVA was used. A two sample *t*-test was applied to compare any significant difference between the current results with other studies, and to determine any difference between the MPs concentration of bottled water and tap water. The data analyzed by flow cytometry were processed using FlowJo 10.7.2, and the general gating method was described in Tse et al. [16].

## 3. Results and Discussions

### 3.1. Abundance and Shapes of Large MPs (≥50 µm)

Large MPs with a size of ≥50 µm in bottled and tap water samples were stained by NR and quantified manually by a stereomicroscope with a blue LED laser light source and an orange emissions filter. According to Table 2, MPs with a size of ≥50 µm were detected in all bottled and tap water samples, where the individual average MPs abundance of the bottled and tap water samples was found ranging from 8–50 particles per L^–1^, with relative standard deviation varying from 0.02 to 0.6%. The overall average MPs abundance of all 12 bottled and tap water samples was found to be 19 ± 14 particles per L^−1^. Mason et al. [13] observed an average of 10 ± 8 particles per L^–1^ for MPs with a size of ≥100 µm from 11 different brands of bottled water, where another study by Kankanige and Babel. [19] reported an average MPs abundance (with a size of ≥50 µm) of 12 ± 6 particles per L^–1^ from 13 PET and glass water bottles. Comparing our observations with these two studies, there was no statistically significant difference (one-way ANOVA, *p* > 0.05).

By comparing the abundance of large MPs among different types of bottled waters (i.e., distilled, spring, and mineral), there was no significant difference among the three bottled types (one-way ANOVA, *p* > 0.05), whereas large MPs in tap water were found to be significantly lower than in mineral water (*p* = 0.026) but no significant difference for tap water with other types of bottled water. Although the average amount of large MPs found in tap water samples (12 ± 8 particles L^–1^) was the lowest when compared with the average of other bottled waters, it was much higher than the mean results from another study of Hong Kong tap water samples by Lam et al. [20] in 2020 (with average MPs of 2.181 ± 0.165 particles L^–1^). In a study by Lam et al. [20], the samples were treated with Rose Bengal dye which stained cellular materials instead of MPs [21], where identification of some MPs, especially those clear in color, may be difficult and caused underestimation of count. The use of NR staining can increase the sensitivity in detecting MPs.

**Figure 1:**
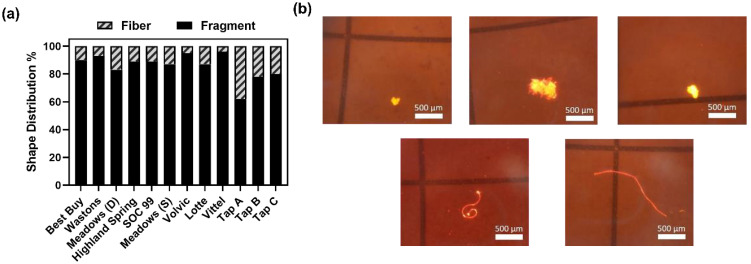
(**a**) The shape distribution (%) of large microplastics (≥50 µm) detected in nine brands of bottled water samples and three sources of tap water samples. The shape of detected large MPs were classified into two types: fiber and fragment; (**b**) microscope views of NR stained MPs found in samples. Scale bar represents a length of 500 µm (excitation: 450 nm; emission 590 nm).

As higher incidents of fiber type MPs are found in lung cancer tissue biopsies [22], the shape of MPs would affect uptake by cells, and it is important to study the shape information of MPs. Figure 1a,b illustrates the shape distribution and microscopic images of some large MPs found in the samples, respectively. Similar to the study of Kankanige and Babel [19], only fiber and fragment types were observed. Fragment was found to be more dominant in all samples, with more than 80% and 60% of the large MPs in fragment shape found in bottled and tap water samples, respectively. The proportions observed were different from the study of Kankanige and Babel [19], where higher proportions of fiber were observed. As proposed by their study, the proportion of fiber would be increased when the size of MPs decreased. The discrepancy in shape distribution may be caused by the size difference between studies. Moreover, it should be noted that the average percentage of fiber found in tap water samples was significantly higher than in bottled water (two sample *t*-test, *p* = 0.0182), reflecting that the processes to remove MPs at drinking water treatment works and bottled water manufacturing faculties may be varied.

### 3.2. Abundance of Small MPs (1–50 µm)

**Figure 2:**
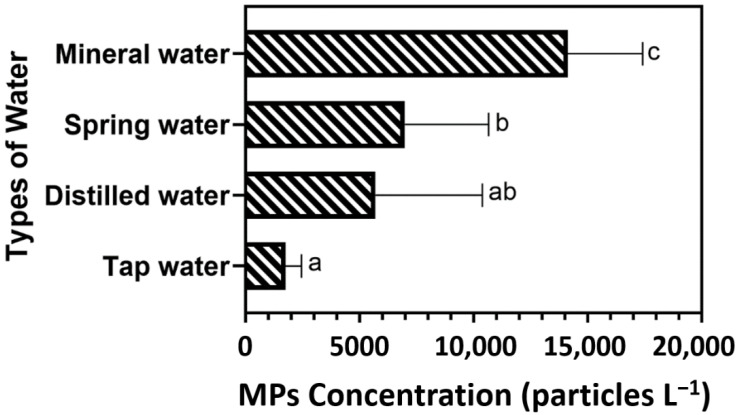
MPs concentration (particles L^–1^) in four water types (distilled water, spring water, mineral water, and tap water). Each bar and error bar represents the mean concentration and standard deviation, respectively. Different letters (a–c) were assigned for representing the statistical differences, same letter represents there was no significant difference (*p* > 0.05), while different letters represent there was significant difference (*p* < 0.05).

To quantify MPs with size ranging from 1–50 µm in the samples, bottled water and tap water samples after pre-concentration, wet oxidation and NR staining were analyzed by the previously developed flow cytometry approach [18]. As shown in Table 2, the average MPs counts varied from 927 ± 497 particles per L^–^^1^ to 17,393 ± 4304 particles per L^–^^1^. As shown in Figure 2, the average concentration of small MPs from different types and origins were plotted and analyzed statistically. The concentration of small MPs in mineral water was significantly higher than distilled water, spring water and tap water samples (one-way ANOVA, *p* < 0.05), indicating that the sources and processing of mineral water may introduce higher contamination of small size MPs. Moreover, there was no significant difference in small MPs between tap and distilled waters (one-way ANOVA, *p* > 0.05), but small MPs in tap water were statistically less than those found in spring and mineral water samples (one-way ANOVA, *p* = 0.0140 and <0.0001 respectively), indicating the manufacturing processes in treating water from underground origins may not be as effective as those for distilled and tap waters in removal of MPs.

Combining MPs in all sizes (from 1 µm to 5mm) within the same sample, the average MPs concentration found in tap water samples was 1753 ± 693 particles per L^–1^, which was significantly lower (two sample *t*-test, *p* = 0.0003) than the average amount of MPs in bottled water samples, which accounted for 8955 ± 5205 particles per L^–1^. Danopoulos et al. [23] reported similar findings of higher MPs contamination levels in bottled water than in tap water, which suggested MPs contamination may appear during the production or packaging process of bottled water, since tap water treatment does not include bottle filling steps. Filtration was often used in water treatment for both bottled and tap water. Yang et al. [24] revealed that ultra-filtration can remove more than 70% of MPs, whereas sand filtration can remove more than 90% of MPs with a size greater than 10 µm [25]. Variations in filtration processes used in bottled water manufacturing may lead to the variation in MPs abundance. Reverse osmosis (RO), another water treatment, can effectively separate water from MPs; however, defects in RO membranes and small openings in system pipework can result in MPs contamination [26].

Furthermore, the average concentration of MPs presented in the bottled water samples of this study did not statistically differ from the results (2649 ± 2857 particles per L^–1^) of Oßmann et al. [14] by the use of micro-Raman spectroscopy (two sample *t*-test, *p* > 0.05), suggesting the use of flow cytometry and micro-Raman spectroscopy produced comparable MPs counts in bottled water samples.

### 3.3. Size Distribution of MPs in Bottled and Tap Water

**Figure 3:**
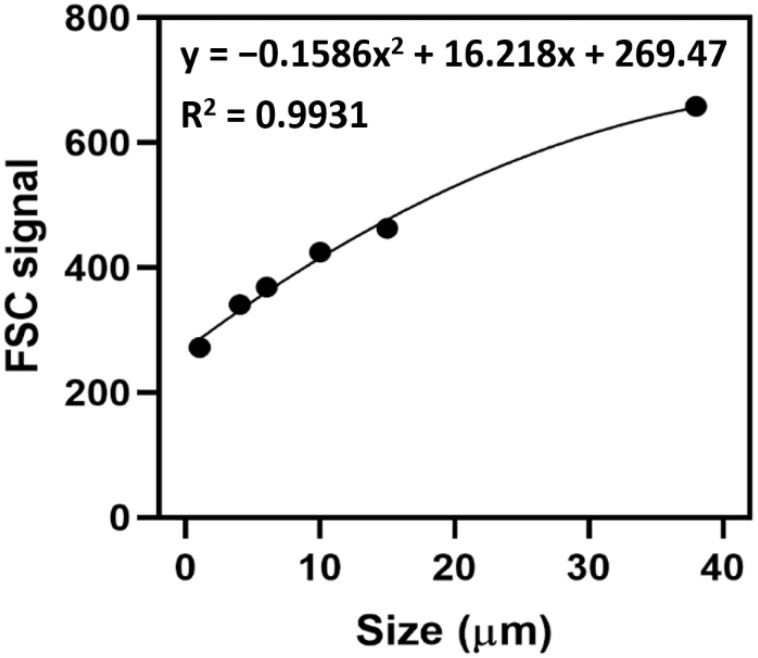
The calibration curve of FSC signal versus reference beads with sizes of 1, 4, 6, 10, 15 and 38 µm.

In order to accurately determine the size of MPs, calibration of forward scatter signals of the flow cytometer using MPs with known size is required. Figure 3 shows the relationship of forward scatter (FSC) signal against NR-stained reference beads with mean size at 1, 4, 6, 10, 15 and 38 µm. A strong correlation (R^2^ = 0.9931) was observed between the FSC signal and the size of MPs under binomial distribution. With the use of this calibration curve, the size of MPs passing through the flow cell can be evaluated, which can facilitate the understanding on the size distribution of MPs in the samples.

**Figure 4:**
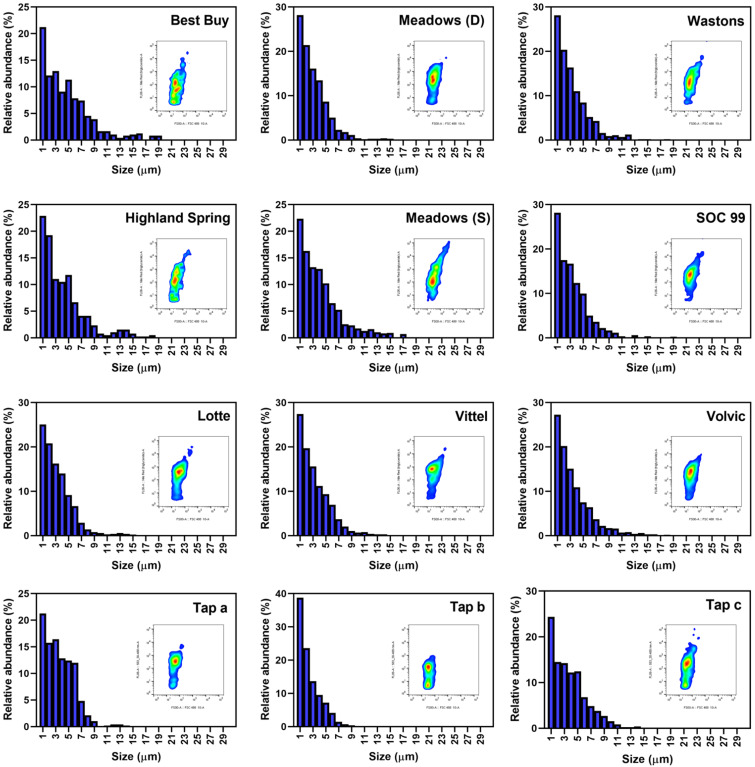
The relative abundance distribution and flow cytometry histogram of small microplastics detected in the bottled and tap water samples.

Figure 4 shows the relative size distribution of detected small MPs in the bottled and tap water samples, together with the corresponding flow cytometry diagrams of NR fluorescence versus FSC signals. The size of all MPs was rounded to micron level. The result clearly showed that smaller MPs have higher abundance, with the modal size at 1 µm for all samples. More than 90% of the relative abundance was contributed by MPs with sizes between 1 and 10 µm. The results aligned with the finding from Eriksen et al. [27], where MPs with smaller size had higher abundance. A similar pattern of results was obtained by Schymanski et al. [28], showing that MPs with a size of 5–10 µm (39–56%) were significantly more abundant than those larger than 100 µm (1–7%). By the use of flow cytometry, a more detail size distribution of small MPs with resolution down to 1 µm can be provided.

**Figure 5:**
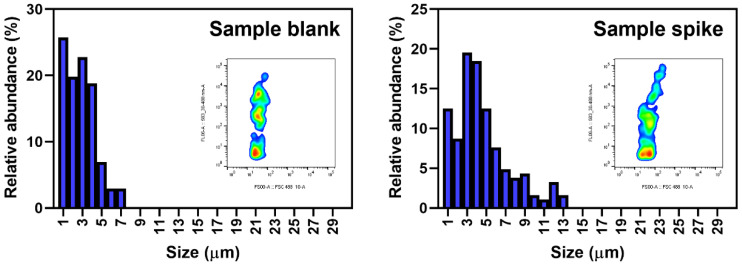
The relative abundance distribution and flow cytometry histogram of small microplastics detected in sample blank and sample spike.

Figure 5 shows the relative size distribution of detected small MPs in the recovery study to assess the efficiency in quantifying stained MPs by flow cytometry in the presence of a drinking water matrix. The size of all MPs was rounded to micron level. The average amount of stained MPs in the sample blank and sample spike by the flow cytometry was found to be 1200 ± 310 particles and 2380 ± 76 particles, respectively. With an amount of 1158 ± 378 PET powder fortified to the sample spike, the recovery of PET was found to be 101 ± 6.6%. No significant loss of stained PET signal was found (two sample *t*-test, *p* > 0.05). The modal size of MPs in sample blank and sample spike was found at 1 µm and 3 µm, respectively, where the increase in modal size of MPs in the sample spike was contributed by a larger size of PET (average size at 4.9 µm) fortified. The results were found to be similar to our previous study spiking other plastic types in ultrapure and seawater samples [18].

### 3.4. Assessment of Potential MPs Contamination and Interference

**Figure 6:**
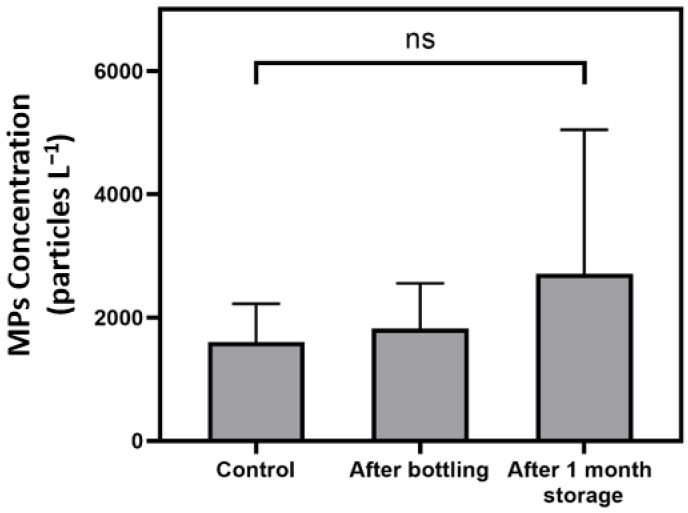
MPs concentration (particles L^−1^) in control (ultrapure water), immediately after the bottling and after 1 month of storage at room temperature conditions after bottling. Each bar and error bar represents the mean concentration and standard deviation, respectively. The label ns indicates there are no significant differences (*p* > 0.05) of the samples.

Gambino et al. [7] proposed some sources of MPs contamination to bottled water, including the filling processes and storage conditions. To determine the significance of bottle filling and storage to small MPs contaminations, experiments were carried out to determine any increase in MPs abundance by filling of ultrapure water to single-use PET bottles. MPs determination was conducted immediately after bottling and after 1-month storage at room conditions, and the MPs abundance was compared with those in ultrapure water (control). Figure 6 summarizes the small MPs found in control, after filling and after 1-month storage. Although there were slightly increases in the average concentration and variation after the filling and storage, there was no significant difference with the control (one-way ANOVA, *p* = 0.6361). However, slightly higher average concentration found in filling and storage indicated that MPs were inherently contaminated in small amounts, and the level of MPs may be further increased for longer storage duration. Weisser et al. [29] conducted a study to evaluate the potential sources of MPs contamination in mineral bottled water during the processing steps, where the processes of filling and capping were identified as the major cause of MPs contamination. Moreover, opening and re-capping of bottles would generate MPs due to abrasion between the cap and bottleneck [30].

On the other hand, the small MPs abundance in the experiments was found to be significantly lower than the commercial bottled water samples (two sample *t*-test, *p* = 0.0066), implying that there may be other contamination sources during the water treatment stage.

**Figure 7:**
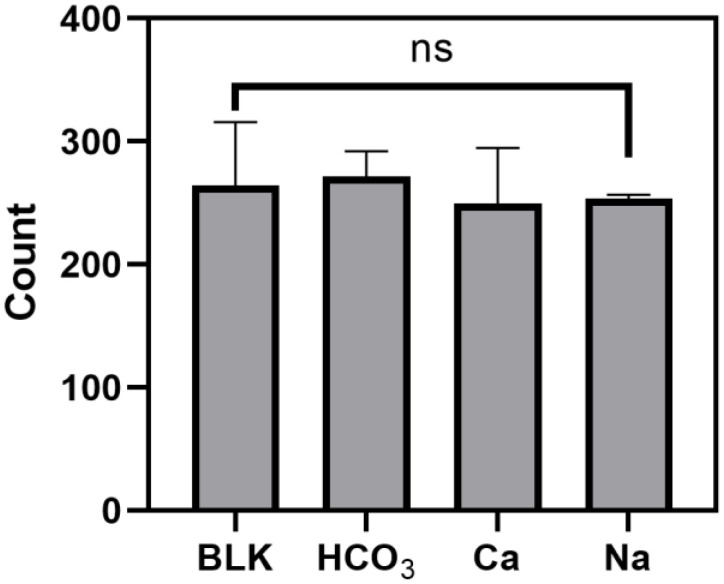
Result of flow cytometry count of the control (ultrapure water), and ultrapure waters fortified with bicarbonate (HCO_3_^−^) at a concentration of 380 mg per L^−1^, calcium (Ca^2+^) at a concentration of 240 mg per L^−1^, and sodium (Na^+^) at a concentration of 50 mg per L^−1^. Each bar and error bar represents the mean concentration and standard deviation, respectively. The label ns indicates no significant differences (*p* > 0.05) between different samples.

In addition, any potential interference caused by additives, such as HCO_3_^−^, Ca^2+^ and Na^+^, to the flow cytometry signal was evaluated. Figure 7 indicates the MPs abundance in control (ultrapure water), and ultrapure water fortified with HCO_3_^−^, Ca^2+^ and Na^+^, where no significant differences between samples with and without adding of salts (one-way ANOVA, *p* = 0.8672), indicating that the presence of HCO_3_^−^, Ca^2+^ and Na^+^, at concentrations benchmarked with the market, would not cause any interference to the FSC signal. It is worth noting that only mineral water involves the process of minerals addition, which can be a possible source of MPs contamination as various studies have shown that rock salts with rich mineral content were polluted by MPs [31,32].

### 3.5. Estimation of MPs Intake by Humans

By the use of the MPs abundance results, the estimated daily intake (EDI) of MPs from drinking water was evaluated using the following equation:

EDI = (C × IR)/bw(2)

where C, IR and bw represent the concentration of MPs (particles per L^–1^) detected, ingestion rate (L day^–1^) and body weight of a human. According to the statistical data, the average volumes of bottled water and drinking water consumption in Hong Kong were 0.310 L per day^–1^ [33] and 1.179 L per day^–1^ [34] respectively. The IR values of bottled and tap waters were thus assigned as 0.310 L per day^–1^ and 0.869 L per day^–1^, respectively, with the assumption that bottled and tap waters are the only sources of drinking water. The average body weight (kg) of Hong Kong people with age ranging from 15–84 was 62.3 kg [35]. As shown in Table 3, the calculated EDI for large MPs in bottled and tap waters were 0.109 particles (kg·d)^–1^ and 0.167 particles (kg·d)^–1^, respectively. The total of EDI for large MPs from drinking water in Hong Kong (0.276 particles (kg·d) ^–1^) was found to be similar to another study in China (0.274 particles (kg·d)^–1^) [12]. Samandra et al. [36] analyzed MPs with sizes between 20–500 μm from 16 brands of bottled water sold in Australia, where the exposure of MPs found (400 particles per year) was at least 15 times lower than large MPs of this study, implicating a large variation of exposure to large MPs from bottled water in different parts of the world.

However, by considering together with the EDI of small MPs in bottled water and tap water (i.e., 44.5 particles (kg·d)^–1^ and 24.5 particles (kg·d)^–1^, respectively), the total EDI of MPs at all sizes from drinking water was found to be 69.2 particles (kg·d)^–1^, which is 10 times more than the findings from Cox et al. [37] by the use of data from the United States. Previously the exposure was underestimated by considering MPs with larger sizes due to quantitative method limitations. From the EDI results, the exposure of MPs through drinking water is prevalent. With the aid of flow cytometry, small MPs with sizes ranging from 1–50 µm can be accurately measured and clear exposure data can be obtained. Indeed, to reduce the exposure of MPs from drinking water, individuals should consume less bottled water and drink more tap water.

## 4. Conclusions

Accurately determining the occurrence and size distribution of full-size MPs in drinking water, including bottled and tap waters, is crucial to estimate the level of MPs exposure in human diets. Using both fluorescence microscopy and flow cytometry, the abundance of full-size MPs in bottled and tap water samples can be determined and the MPs abundance in the bottled and tap waters were found to be significantly higher than other studies focusing on MPs in larger sizes. With calibration of the FSC signal from flow cytometry, size measurement and distribution of MPs can be provided. Small MPs with sizes 1–10 µm (average 6827 ± 5483 particles per L^–1^) were found to be more abundant in all samples when compared to larger MPs (334 ± 236 and 19 ± 14 particles per L^–1^ for sizes 10–50 µm and 50 µm–5 mm, respectively), and MPs with sizes 1–10 µm that may penetrate through biological barriers were found as the majority in abundance in all samples. The concentration of small MPs (1–50 µm) in mineral water was significantly higher than other types of drinking water, and small MPs in tap water were statistically less than with those found in spring and mineral water samples, indicating the manufacturing processes in removing MPs in water from underground origins may not be as effective as the tap and distilled waters. Although there is a slight increase in the average concentration of MPs during the filling and storage, the major source of MPs contamination in bottled water was found to be contributed by the water treatment stage before filling.

By evaluating the EDI of full-size MPs, the exposure to MPs from drinking water (i.e., 69.2 particles (kg·d)^–1^) was underestimated when studies only considered large MPs. The findings in this study, can be used as a reference to set baseline levels of MPs from drinking water, for further exposure and toxicological studies of MPs on humans. More studies are required to investigate full size MPs from other foods so as to provide a better estimation of MP exposure in humans. Nevertheless, humans are exposed to substantial amounts of MPs from drinking water; it is still unknown the risk to human health by consuming of this level of MPs. With a better knowledge on the type, size, shape and abundance of MP exposure, risk assessments of MPs to public health can be evaluated and mitigating measures to reduce the potential risk can be formulated.

## Figures and Tables

**Table 1:** Characteristics of nine bottled water samples, namely brand, origin, water type, volume per liter and material of bottle.

Sample	Brand	Origin	Water Type	Volume (L)	Material of Bottle
1	Best Buy	Hong Kong	Distilled water	6	PET
2	Watsons	Hong Kong	Distilled water	4.5	Recycle PET
3	Meadows	Malaysia	Distilled water	1.5	PET
4	SOC 99	Japan	Spring water	2	PET
5	Highland Spring	Scotland	Spring water	1.5	PET
6	Meadows	France	Spring water	1.5	PET
7	Volvic	France	Mineral water	1.5	PET
8	Vittel	France	Mineral water	1.5	Recycle PET
9	Lotte	Korea	Mineral water	2	PET

**Table 2:** MPs concentration (particles L^–1^) and average relative abundance (ARA %) in nine brands of bottled water and three sources of tap water with different size ranges, namely 1–10 µm, 10–50 µm and ≥50 µm. Data presented were mean values and standard deviation from triplicates.

Water Samples	1–10 µm	ARA %	10–50 µm	ARA %	≥50 µm	ARA %
Best Buy	1260 ± 883	80.5 ± 56.4	295 ± 280	18.8 ± 17.9	11 ± 2	0.7 ± 0.1
Watsons	4667 ± 1620	98.4 ± 34.1	48 ± 114	1.0 ± 2.4	29 ± 19	0.6 ± 0.4
Meadows (D)	10,640 ± 3187	98.2 ± 29.4	188 ± 42	1.7 ± 0.4	11 ± 3	0.1 ± 0.03
SOC 99	9707 ± 2290	97.8 ± 23.1	202 ± 170	2.0 ± 1.7	12 ± 2	0.1 ± 0.02
Highland Spring	2660 ± 2393	93.5 ± 84.1	175 ± 92	6.2 ± 3.2	9 ± 5	0.3 ± 0.2
Meadows (S)	7600 ± 2754	91.6 ± 33.2	668 ± 304	8.1 ± 3.7	27 ± 5	0.3 ± 0.1
Volvic	11,060 ± 5669	95.4 ± 48.9	488 ± 201	4.2 ± 1.7	50 ± 21	0.4 ± 0.2
Lotte	17,393 ± 4304	97.2 ± 24.1	488 ± 232	2.7 ± 1.3	8 ± 4	0.04 ± 0.02
Vittel	12,787 ± 7988	97.3 ± 60.8	315 ± 251	2.4 ± 1.9	39 ± 11	0.3 ± 0.1
Tap water A	1533 ± 1094	82.8 ± 59.1	300 ± 100	16.2 ± 5.4	18 ± 11	1.0 ± 0.6
Tap water B	1693 ± 488	97.2 ± 28.0	40 ± 40	2.3 ± 2.3	9 ± 3	0.5 ± 0.2
Tap water C	927 ± 497	53.4 ± 28.6	800 ± 20	46.1 ± 1.2	9 ± 5	0.5 ± 0.3
Average	6827 ± 5483	95.1 ± 76.4	334 ± 236	4.7 ± 3.3	19 ± 14	0.3 ± 0.2

**Table 3:** Calculated EDI of MPs in bottled water and tap water in Hong Kong.

	Hong Kong (Bottled Water)	Hong Kong (Tap Water)	Hong Kong (Total)
IR (L day^−1^)	0.310	0.869	1.179
bw (kg)	62.3		
Average concentration of ≥50 µm MPs (MPs L^−1^)	22	12	-
Average concentration of ≥25 µm MPs (MPs L^−1^)	34	28	-
Average concentration of <50 µm MPs (MPs L^−1^)	8934	1753	-
Calculated EDI of ≥50 µm MPs	0.109	0.167	0.276
Calculated EDI of <50 µm MPs	44.455	24.463	68.918
Calculated EDI of 1 µm to 5 mm MPs	44.564	24.630	69.194

## Data Availability

Not applicable.
